# Supplementary material for: The causal role of circulating inflammatory markers in osteoporosis: a bidirectional Mendelian randomized study
Source: Front Immunol. 2024 Jul 18;15:1412298. doi: 10.3389/fimmu.2024.1412298 (PMC11291241; doi:10.3389/fimmu.2024.1412298)
Supplement: Supplementary file 1 [file DataSheet_1.zip › supplementary material/OSTEOPOROSIS-CIM/GCST90274783.tsv.gz_leave_one_out.pdf]

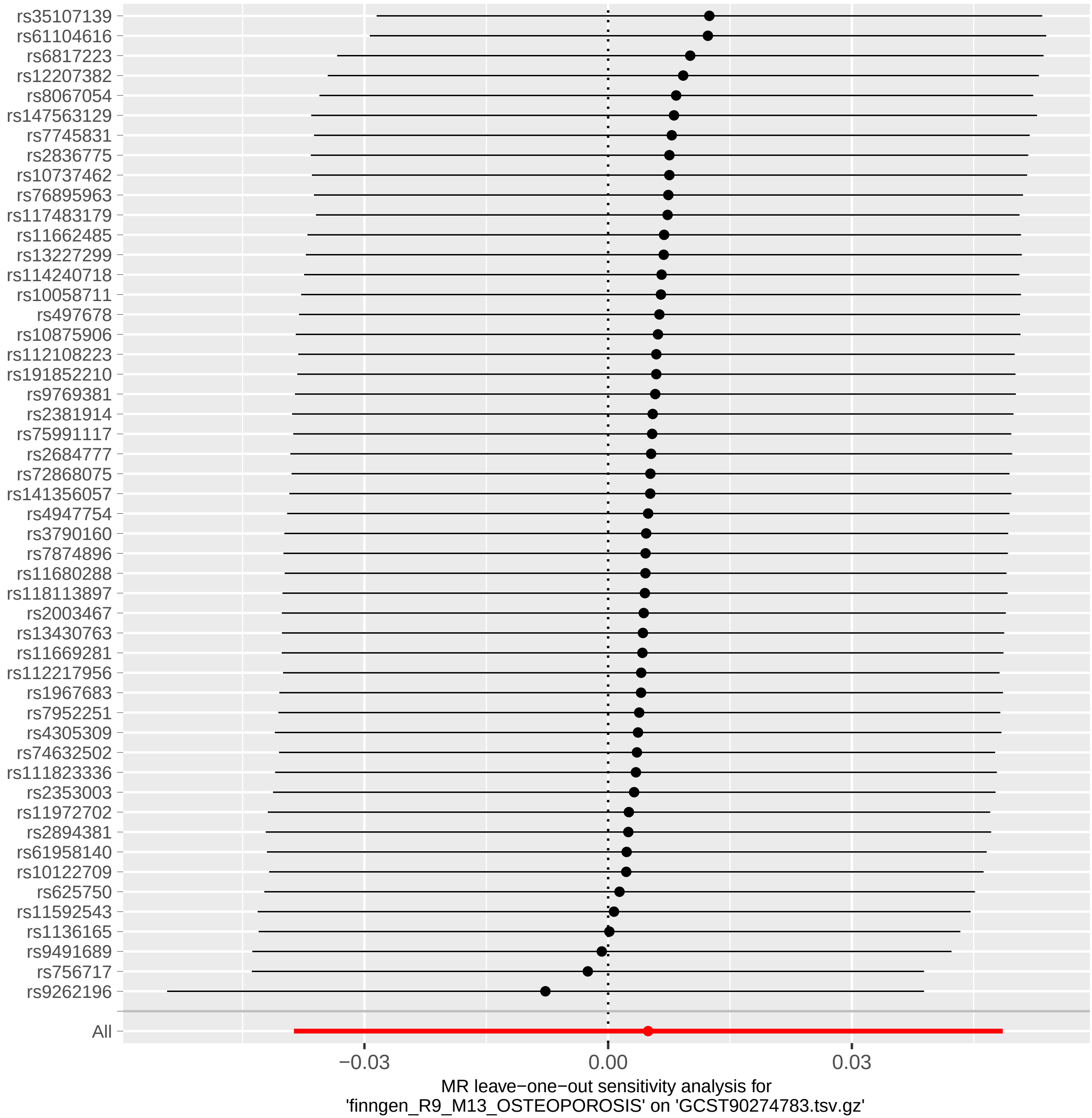
rs35107139
rs61104616
rs6817223
rs12207382
rs8067054
rs147563129
rs7745831
rs2836775
rs10737462
rs76895963
rs117483179
rs11662485
rs13227299
rs114240718
rs10058711
rs497678
rs10875906
rs112108223
rs191852210
rs9769381
rs2381914
rs75991117
rs2684777
rs72868075
rs141356057
rs4947754
rs3790160
rs7874896
rs11680288
rs118113897
rs2003467
rs13430763
rs11669281
rs112217956
rs1967683
rs7952251
rs4305309
rs74632502
rs111823336
rs2353003
rs11972702
rs2894381
rs61958140
rs10122709
rs625750
rs11592543
rs1136165
rs9491689
rs756717
rs9262196
All
−0.03
0.00
0.03
MR leave−one−out sensitivity analysis for
'finngen_R9_M13_OSTEOPOROSIS' on 'GCST90274783.tsv.gz'
